# A new parameter in the era of robotic total knee arthroplasty: Coronal alignment at 90° of flexion impacts clinical outcomes

**DOI:** 10.1002/ksa.12648

**Published:** 2025-03-18

**Authors:** Luca Andriollo, Christos Koutserimpas, Pietro Gregori, Elvire Servien, Cécile Batailler, Sébastien Lustig

**Affiliations:** ^1^ Orthopaedics Surgery and Sports Medicine Department, FIFA Medical Center of Excellence, Croix Rousse Hospital, Hospices Civils de Lyon Lyon North University Hospital Lyon France; ^2^ Sezione di Chirurgia Protesica ad Indirizzo Robotico ‐ Unità di Traumatologia Dello Sport, Ortopedia e Traumatologia, Fondazione Poliambulanza Istituto Ospedaliero Brescia Italy; ^3^ Ortopedia e Traumatologia Università Cattolica del Sacro Cuore Rome Italy; ^4^ Fondazione Policlinico Universitario Campus Bio‐Medico Roma Italy; ^5^ LIBM‐EA 7424, Interuniversity Laboratory of Biology of Mobility Claude Bernard Lyon 1 University Lyon France; ^6^ Univ Lyon, Claude Bernard Lyon 1 University, IFSTTAR, LBMC UMR_T9406 Lyon France

**Keywords:** alignment in flexion, dynamic HKA, functional alignment, functional knee positioning, knee arthroplasty, TKA

## Abstract

**Purpose:**

Alignment strategies in total knee arthroplasty (TKA) have predominantly emphasized coronal alignment in extension, with minimal focus on dynamic alignment during flexion. This study aims to identify the predictors of the intraoperative robotic hip–knee–ankle angle at 90° of flexion (rHKA‐90F) and assess its clinical significance in postoperative outcomes, proposing that rHKA‐90F may be a critical factor in enhancing functional results in TKA.

**Methods:**

A retrospective analysis was conducted on 180 patients with varus deformity undergoing robotic‐assisted TKA under the functional alignment principles. Clinical outcomes were assessed using the Knee Society Score (KSS), Forgotten Joint Score (FJS‐12) and Kujala Anterior Knee Pain Scale (AKPS) score. Predictors for final rHKA‐90F and its intraoperative changes were identified using multiple linear regression models. Initial and intraoperative robotic measurements were also analyzed.

**Results:**

Significant predictors of the final rHKA‐90F included femoral rotation, tibial varus/valgus alignment, initial rHKA‐90F and the final robotic axis in extensions. Patients with a final rHKA‐90F ≥ 5° of varus demonstrated superior KSS function and KSS knee compared to those with a final rHKA‐90F between 0° and 4° of varus. Furthermore, patients with intraoperative changes of rHKA‐90F > 2.5° neutralization (varus reduction or with a valgus value) achieved better FJS and AKPS score.

**Conclusions:**

This study highlights the clinical relevance of rHKA‐90F as an intraoperative tool in robotic knee arthroplasty, emphasizing the need to balance the correction of varus deformity with the maintenance of slight varus alignment in flexion. Personalized alignment strategies tailored to patient‐specific anatomy and kinematics are crucial to optimizing outcomes. There is still a need for future research on the long‐term effects of dynamic alignment.

**Level of Evidence:**

Level III.

**Abbreviations**
aHKA: arithmetic hip–knee–ankle angle
AKPS: Kujala Anterior Knee Pain Scale
BMI: body mass index
CPAK: Coronal Plane Alignment of the Knee
CS: cruciate substituting
FA: functional alignment
FJS‐12: Forgotten Joint Score
FKP: functional knee positioning
HKA: hip–knee–ankle angle
JLO: joint line obliquity
KSS: Knee Society Score
LDFA: lateral distal femoral angle
MCID: minimal clinically important difference
mHKA: mechanical hip–knee–ankle angle
MPTA: medial proximal tibial angle
PROM: patient‐reported outcome measure
PS: posterior stabilized
rHKA‐90F: robotic HKA at 90° of flexion/coronal alignment at 90° of knee flexion
rHKA‐E: robotic hip–knee–ankle angle in full extension
ROM: range of motion
SD: standard deviation
TKA: total knee arthroplasty
ΔKSS: difference between final and preoperative KSS knee score
ΔrHKA‐90F: difference between final and initial rHKA‐90F

## INTRODUCTION

Total knee arthroplasty (TKA) aims to achieve optimal limb alignment. However, studies on varus correction and the significance of residual varus have so far been based on the hip–knee–ankle angle (HKA) in extension, primarily measured on full‐length weight‐bearing x‐rays. The advent of robotic‐assisted TKA has revolutionized the field, providing the ability to gain insights into previously unquantifiable intraoperative parameters [1, 8, 22]. The alignment in flexion is correlated with knee functionality, which is a dynamic joint [24]. It is not a parameter strictly related to the HKA in extension. Only a small percentage of knees (14.1%) maintain a consistent alignment throughout flexion, while the majority exhibit changes in deformity or even reversal during the flexion range [6].

This study introduces the concept of intraoperative robotic measurements to assess coronal alignment at 90° of knee flexion, referred to as the robotic HKA at 90° of flexion (rHKA‐90F). This novel concept could enhance the understanding of knee kinematics, particularly in terms of how alignment transitions from preoperative deformity to postoperative correction.

Factors influencing the final rHKA‐90F and its connection to initial alignment must be understood to optimize surgical outcomes. Furthermore, investigating whether rHKA‐90F can reliably predict postoperative function and kinematics is essential for advancing clinical practices. Additionally, understanding how variations between initial and final rHKA‐90F affect clinical outcomes will provide valuable insights into patient recovery and overall success rates. The objective of this study is to investigate the predictors of final rHKA‐90F and its intraoperative changes. Additionally, the study aimed to explore whether the final rHKA‐90F or its difference from the initial state influences clinical outcomes. The hypothesis was that rHKA‐90F may play a role in knee kinematics and that understanding it could improve postoperative outcomes in TKA.

## METHODS

This retrospective study was conducted using a prospectively maintained database. It included all patients undergoing primary TKA, under the principles of functional knee positioning (FKP), an image‐based robotic system between March 2021 and December 2022. The same single‐radius prosthetic implant was used in all patients, with either posterior stabilized (PS) or cruciate substituting (CS) inserts.

All procedures were performed at a single high‐volume centre specializing in both primary and revision arthroplasty surgeries. All patients had a minimum follow‐up period of two years, and their medical records, imaging studies, and outpatient consultations were reviewed by two investigators (LA and CK).

Patients with a mechanical HKA (mHKA) angle less than or equal to 180° on preoperative full‐length weight‐bearing x‐rays were included. A mHKA angle greater than 180° was considered an exclusion criterion (87 patients). Patients were excluded if their preoperative, intraoperative or follow‐up data were incomplete or if a detailed robotic data report was missing (114 patients). Additionally, patients requiring revision surgery were excluded (one for femoral component revision, one for acute infection and one for complete revision due to an undiagnosed nickel allergy). Therefore, 180 patients were included.

At the final follow‐up, 180 patients were evaluated. Among these, 80 were male (44.4%) and 100 were female (55.6%). The average age at the time of surgery was 69.1 ± 8.3 years. The surgical side was the left in 87 cases (48.3%) and the right in 93 cases (51.7%). The mean body mass index (BMI) was 28.9 ± 4.6 kg/m². Preoperative data for all patients included the knee range of motion (ROM), as well as Knee Society Score (KSS) function and knee parts. Imaging evaluations were performed using anteroposterior, lateral, Rosenberg, sunrise, and full‐length weight‐bearing x‐rays. From these, the mHKA, lateral distal femoral angle (LDFA), medial proximal tibial angle (MPTA), arithmetic HKA (aHKA), joint line obliquity (JLO) and preoperative Coronal Plane Alignment of the Knee (CPAK) classification were calculated [17].

Intraoperative data were obtained from robotic system records, which were documented in the surgical reports and archived using intraoperative screenshots. These data included component positioning metrics. For the femoral component, assessments included flexion/extension, varus/valgus alignment of the distal cut and rotation relative to the transepicondylar axis. For the tibial component, varus/valgus alignment of the proximal cut and posterior slope were analyzed.

Preoperative, postoperative, and the resulting differences were recorded, along with the initial and final robotic‐measured HKA in full extension (rHKA‐E), which is the value provided by the robotic system during the intraoperative assessment (before and after the cuts for implant) of the coronal axis. At the final follow‐up, clinical evaluations included KSS‐knee and function, the Forgotten Joint Score (FJS‐12) and the Kujala Anterior Knee Pain Scale (AKPS). In addition, ΔKSS knee and ΔKSS function were calculated, representing the difference between the preoperative value and the value at the final follow‐up.

**Figure 1:**
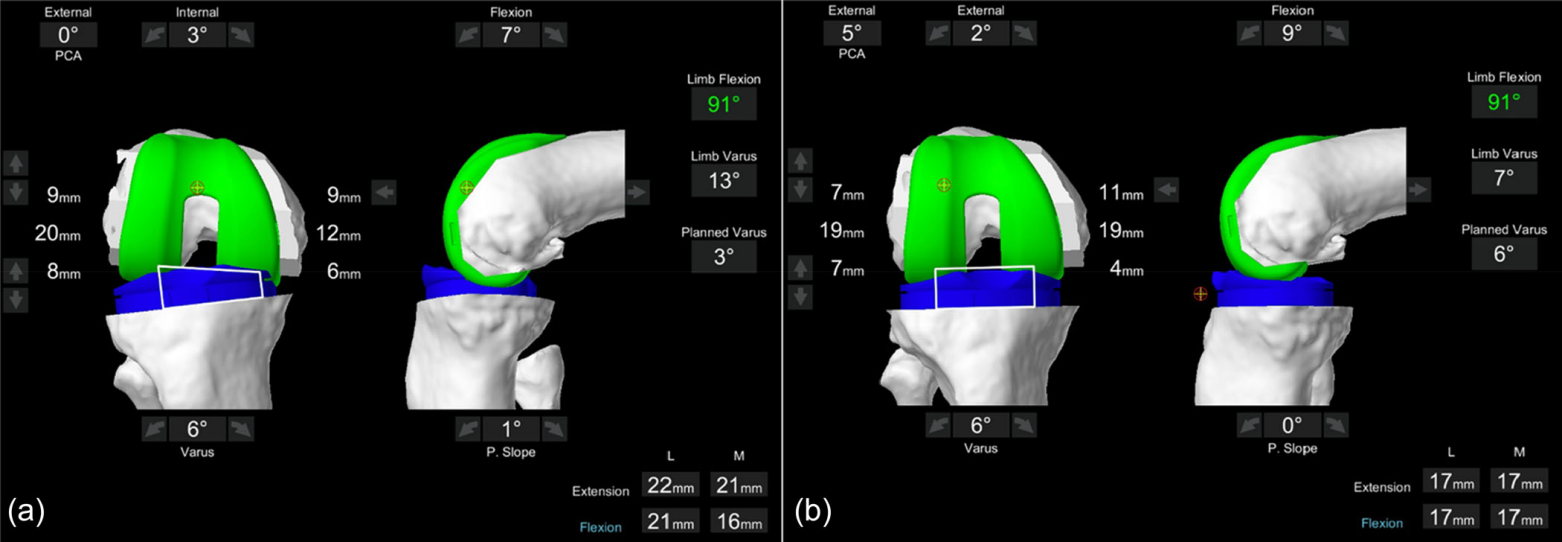
Intraoperative screenshots from the Mako robotic arm‐assisted system (Stryker, Mako Surgical Corp.). Image (a) represents the evaluation of the initial limb varus/valgus axis at 90° of flexion (rHKA‐90F), while image (b) shows the evaluation of the final rHKA‐90F in the same patient. Specifically, the patient had an initial rHKA‐90F of 13° of varus, which was corrected to 7° of varus at the end of the procedure. rHKA‐90F, robotic hip–knee–ankle angle at 90° of flexion.

The robotic system calculated the rHKA‐90F as the angle between the projection of the femoral and tibial mechanical axes on the coronal plane. Specifically, the femoral mechanical axis is the line that extends from the hip centre, defined as the centre of the femoral head, to the femur knee centre, defined as the most distal point of the trochlear groove. The tibial mechanical axis is the line that extends from the tibial knee centre, which is the exit point of the tibial shaft axis in the coronal and sagittal planes, to the central point of the intermalleolar line. To obtain the rHKA‐90F during flexion, the system projects these two axes onto the coronal plane and calculates the angle. The values of rHKA‐90F, as well as rHKA‐E, are recorded without applying varus–valgus stress (Figure 1). In this study, the predictors influencing final rHKA‐90F, as well as those affecting ΔrHKA‐90F, defined as the difference between final and initial values, were evaluated. Additionally, the potential correlation between final rHKA‐90F and functional scores at the final follow‐up was evaluated. Patients were grouped to compare those with a final rHKA‐90F ranging from 4° of varus to 0 (Group A: from 0° to 4° varus) against outlier patients (Group B.1: <0° valgus or >4° varus) and later considering only those with varus angles greater than 4° (Group B.2: >4° varus). Moreover, the ΔrHKA‐90F was evaluated in relation to the outcomes at the final follow‐up. Specifically, patients with a final rHKA‐90F of ±2.5° (Group C: from −2.5° to 2.5°) were compared to those with a neutralized value (varus reduction or with a valgus value) greater than 2.5° (Group D: >2.5°).

In the values considered in this study, robotic data for varus alignment are reported as positive (+) and valgus alignment as negative (−). Regarding the femur, external rotation is presented as positive (+) and internal rotation as negative (−), while femoral flexion is reported as positive (+) and femoral extension as negative (−). For the tibia, the posterior slope is presented as positive (+) and the reverse slope as negative (−).

### Mako robot, Triathlon implant and functional alignment (FA)

All patients underwent surgery following a personalized technique [26]. The Mako robotic arm‐assisted system (Stryker, Mako Surgical Corp.) was employed. This system uses preoperative computed tomography (CT) imaging to create a 3D model of the patient's bone structure. Research has demonstrated that it provides greater accuracy compared to manual methods, reduces soft tissue damage and ensures that the surgical plan is executed with approximately 1° precision [11, 14, 28].

All patients received Triathlon Total Knee System implant (Stryker, Mako Surgical Corp.). Aimed at surpassing older designs by the same producer, the implant has a single‐radius construct that assists in mid‐flexion stability, allows for natural movement of the knee joint, and maximizes implant lifetime. The PS insert was preferred in posterior cruciate ligament deficiency or significant flexion contracture cases.

For all patients, FA or FKP was used [26]. For varus patients, surgical reconstruction was individually tailored to restore the natural coronal alignment of the patient to have terminal HKA of between 174° and 180°. Dynamic sagittal alignment was reconstructed within 5° of neutral as well. Implant sizing was carefully adapted to match each patient's anatomy, and laxity of the soft tissues in flexion as well as in extension was maximized by precisely placing the implant within predefined limits. A personalized surgical plan was set on preoperative imaging, followed by a standardized and measurable evaluation of soft tissue laxity in both extension and flexion. As necessary, the placement of implants was varied in all planes to achieve target measures of the gap and to align the limb within preplanned coronal and sagittal ranges. Throughout the procedure, particular attention was given to preserving the anatomy of the anterior compartment.

### Ethical approval

This study adhered to the ethical principles outlined in the 1964 Declaration of Helsinki and complied with HIPAA regulations. Data collection and analysis were conducted in accordance with the MR004 Reference Methodology of the French Commission Nationale de l'Informatique et des Libertés (Ref. 2229975V0). All patients provided legitimate informed consent.

### Statistical analysis

All data were organized in an Excel spreadsheet. A power analysis was conducted using G*Power (*α* = 0.05, *β* = 0.20, medium effect size), identifying 103 patients as the necessary sample size to evaluate the impact of independent variables of the coronal alignment at 90°. Additionally, a minimum of 48 patients per group was determined to be necessary for comparing outcomes at the final follow‐up. The sample size of this study is based on the minimum required size of the subgroups.

Continuous variables were reported as mean and standard deviation (SD), while categorical variables were presented as frequency distributions and percentages.

The Shapiro–Wilk test was used to assess normality. For statistical comparisons, continuous variables between preoperative and follow‐up assessments were analyzed using either the paired *t* test or the Wilcoxon signed‐rank test, depending on whether the data followed a normal distribution. For comparisons between different groups, the *t* test or the Mann–Whitney *U* test was applied, based on the presence or absence of normal distribution. The categorical variables were compared using the chi‐square test.

To assess the relationship between the predictors and the outcome variable, a multiple linear regression model was employed. A predictor was considered statistically significant if its 95% confidence interval (CI) did not include 0, indicating a consistent positive or negative effect on the outcome variable.

The CI was set at 95%, and statistical significance was defined as a *p* value < 0.05.

To define the minimal clinically important difference (MCID), the distribution‐based SD method was used, considering the MCID as equivalent to 0.5 × SD. Statistical analyses were conducted using Python version 3.11 (Python Software Foundation) with the stats models library (v0.13).

## RESULTS

Table 1 presents the data on preoperative clinical and radiographic evaluation. According to the CPAK classification, the preoperative distribution was as follows: Type I, 74 patients (41.1%); Type II, 43 patients (23.9%); Type IV, 39 patients (21.7%); Type V, 18 patients (10%); Type VI, 2 patients (1.1%); Type VII, 2 patients (1.1%); and Type III, 1 patient (0.6%) [17].

**Table 1:** Preoperative clinical scores, radiographic evaluation and robotic data on implant positioning.

	Average	SD
Preoperative clinical and radiographic evaluation		
KSS‐knee	64.7	12.6
KSS‐function	65.7	16.9
mHKA (degrees)	173.2	3.9
LDFA (degrees)	89.1	5.8
MPTA (degrees)	86.0	2.8
aHKA (degrees)	−3.1	3.5
JLO (degrees)	174.1	13.7
Robotic data on implant positioning		
Tibial varus (degrees)	3.4	1.7
Tibial slope (degrees)	0.7	0.8
Femoral valgus (degrees)	0.5	2.0
Femoral flexion (degrees)	6.6	2.7
Femoral external rotation (degrees)	0.2	1.7

Abbreviations: aHKA, arithmetic hip–knee–ankle angle; JLO, joint line obliquity; KSS, Knee Society Score; LDFA, lateral distal femoral angle; mHKA, mechanical hip‐knee‐ankle angle; MPTA, medial proximal tibial angle.

**Figure 2:**
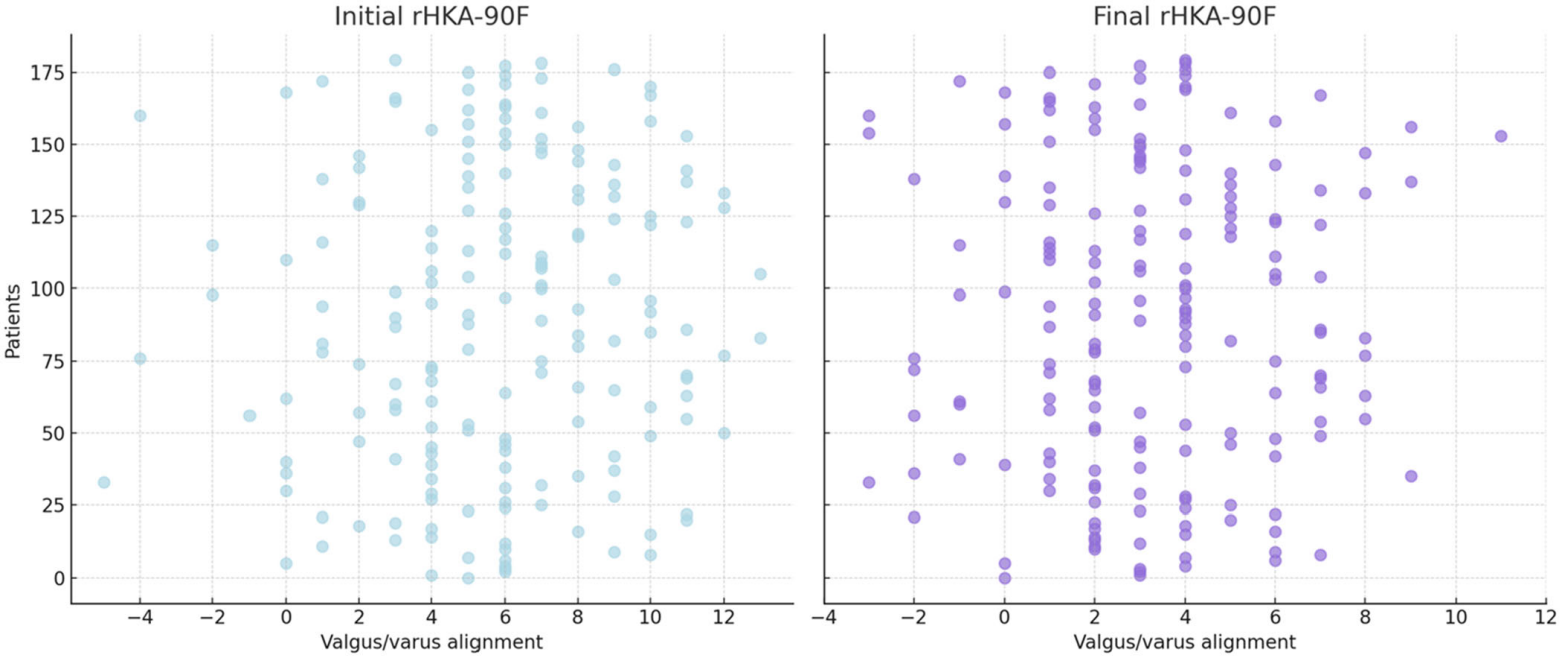
Dot plot showing the distribution of initial and final values of the limb varus/valgus axis at 90° of flexion (rHKA‐90F). Negative degree values correspond to valgus, while positive values correspond to varus. rHKA, robotic hip–knee–ankle angle.

A total of 137 PS implants (76.1%) with PS inserts and 43 CS inserts (23.9%) were used on cruciate retaining implants. Additionally, Table 1 includes robotic data on implant positioning. The mean initial rHKA‐90F was 5.4 ± 3.7°, while the mean final value was 3.2 ± 2.7°, with a statistically significant difference (*p* < 0.001). In 95 patients (52.8%), the rHKA‐90F was neutralized (varus reduction or with a valgus value) by more than 2.5°; in 78 patients (43.3%), it was maintained within an axis of ±2.5°, and in 7 patients (3.9%) the varus alignment was increased by more than 2.5°. Figure 2 represents the graph of the initial rHKA‐90F and final rHKA‐90F values.

At the final mean follow‐up of 2.9 ± 0.6 years, the mean KSS‐knee was 92.7 ± 8.7, the KSS‐function was 91.7 ± 10.3, the FJS‐12 was 74.8 ± 22.4 and the AKPS was 88.4 ± 14.1.

### Predictors of rHKA‐90F

The multiple linear regression analysis identified four variables as significant predictors of the final rHKA‐90F (Table 2). Femoral rotation emerged as a strong positive predictor (*p* < 0.001), indicating that an increase in external femoral rotation is associated with a higher final rHKA‐90F, leading to varus alignment. Similarly, tibial varus/valgus showed a positive association (*p* < 0.001), suggesting that an increase in tibial varus alignment significantly contributes to a higher final rHKA‐90F, also resulting in a more varus alignment. Initial rHKA‐90F (*p* < 0.001) and final rHKA‐E (*p* < 0.001) demonstrated a significant relationship. For these last two predictors, a varus alignment is associated with a greater varus alignment of the final rHKA‐90F.

**Table 2:** Multiple linear regression analysis to identify predictors of coronal alignment at 90° of flexion (rHKA‐90F).

Predictor	Coefficient	Standard error	*t*	*p*	95% CI
Intercept	−0.874	0.490	−1.784	0.076	[−1.840 to 0.093]
Femoral flexion	0.035	0.049	0.708	0.480	[−0.062 to 0.132]
Femoral varus/valgus	0.177	0.112	1.587	0.114	[−0.043 to 0.397]
Femoral rotation	0.582	0.081	7.149	<0.001	[0.421–0.742]
Tibial slope	−0.305	0.167	−1.827	0.069	[−0.635 to 0.025]
Tibial varus/valgus	0.435	0.125	3.478	<0.001	[0.188–0.683]
Initial rHKA‐E	0.009	0.059	0.150	0.881	[−0.107 to 0.124]
Initial rHKA‐90F	0.186	0.049	3.792	<0.001	[0.089–0.283]
Final rHKA‐E	0.383	0.115	3.336	0.001	[0.156–0.609]

Abbreviations: CI, confidence interval; rHKA‐90F, robotic hip–knee–ankle angle at 90° of flexion.

### Predictors of ΔrHKA‐90F

Multiple linear regression analysis was used to identify predictors of ΔrHKA‐90F (Table 3). The only predictor demonstrating a statistically significant influence on ΔrHKA‐90F was tibial varus/valgus, which emerged as a significant negative predictor (*p* = 0.020), indicating that an increase in tibial varus alignment is associated with a lower ΔrHKA‐90F.

**Table 3:** Multiple linear regression analysis to identify predictors of the difference between final and initial limb varus/valgus axis at 90° of flexion (ΔrHKA‐90F).

Predictor	Coefficient	Standard error	*t*	*p*	95% CI
Intercept	−2.058	0.72	−2.859	0.005	[−3.479 to −0.637]
Femoral flexion	0.126	0.081	1.549	0.123	[−0.035 to 0.287]
Femoral varus/valgus	0.038	0.112	0.34	0.734	[−0.182 to 0.258]
Femoral rotation	−0.118	0.12	−0.983	0.327	[−0.354 to 0.118]
Tibial slope	0.011	0.276	0.04	0.968	[−0.533 to 0.555]
Tibial varus/valgus	−0.297	0.126	−2.352	0.02	[−0.546 to −0.048]

Abbreviations: CI, confidence interval; rHKA, robotic hip–knee–ankle angle; ΔrHKA‐90F, difference between final and initial rHKA at 90° of knee flexion.

### Role of rHKA‐90F in outcomes

Clinical outcomes were evaluated between patients with a final rHKA‐90F ranging from 0° to 4° of varus (Group A) and the group excluded from this range (Group B.1). Before comparing outcomes, the homogeneity of the two groups was assessed. The two groups were found to be homogeneous across all evaluated parameters, particularly regarding the type of insert used (CS: 24.8% vs. 22.4%, *p* = 0.71), gender (female: 57.5% vs. 52.2%, *p* = 0.54), age (69.7 vs. 68.7 years, *p* = 0.45), BMI (29.4 vs. 27.9 kg/m², *p* = 0.057), duration of final follow‐up (2.83 vs. 2.87 years, *p* = 0.34), preoperative KSS knee score (65.3 vs. 62.8; *p* = 0.06) and preoperative KSS function score (64.6 vs. 67.1; *p* = 0.37).

**Figure 3:**
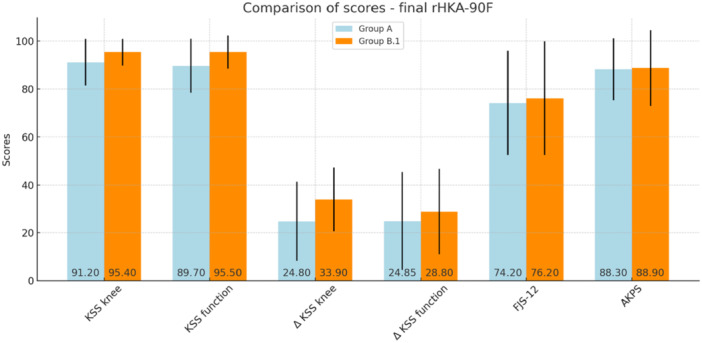
Chart of scores at the final follow‐up between groups with final limb varus/valgus axis at 90° of flexion (rHKA‐90F) within 0–4° of varus (Group A) and those with final rHKA‐90F outside this range (Group B.1). AKPS, Kujala Anterior Knee Pain Scale; FJS‐12, Forgotten Joint Score; KSS, Knee Society Score; rHKA‐90F, robotic hip–knee–ankle angle at 90° of flexion; SD, standard deviation.

A statistically significant difference emerged in terms of KSS function and KSS knee (*p* < 0.001 and *p* = 0.006, respectively), as well as in ΔKSS knee (*p* < 0.001), in favour of the group of patients with final rHKA‐90F not between 0° and 4° of varus. The difference between Groups A and B.1 exceeded the MCID in KSS function and ΔKSS knee (Table 4). The chart of the performed analysis is shown in Figure 3. To exclude a potential influence of patients with a final rHKA‐90F in valgus, the analysis was repeated after excluding them. Patients with a final rHKA‐90F ranging between 0° and 4° of varus (Group A) were compared with those having a final rHKA‐90F ≥ 5° of varus (Group B.2).

**Table 4:** Comparison of scores at the final follow‐up between groups with final limb varus/valgus axis at 90° of flexion (rHKA‐90F) within 0–4° of varus (Group A) and those with final rHKA‐90F outside this range (Group B.1).

	Group A (*N* = 113)	Group B.1 (*N* = 67)	*p*	MCID	Delta (A and B.1)
KSS knee	91.2 (SD 9.7)	95.4 (SD 5.5)	0.006	4.4	4.2
KSS function	89.7 (SD 11.3)	95.5 (SD 6.9)	<0.001	5.2	5.8
ΔKSS knee	24.8 (SD 16.5)	33.9 (SD 13.2)	<0.001	7.7	9.1
ΔKSS function	24.9 (SD 20.4)	28.8 (SD 17.8)	0.113	9.7	3.9
FJS‐12	74.2 (SD 21.7)	76.2 (SD 23.7)	0.267	11.2	2.0
AKPS	88.3 (SD 12.9)	88.9 (SD 15.8)	0.203	7.1	0.6

Abbreviations: AKPS, Kujala Anterior Knee Pain Scale; FJS‐12, Forgotten Joint Score; KSS, Knee Society Score; MCID, minimal clinically important difference; rHKA‐90F, robotic hip–knee–ankle angle at 90° of flexion; SD, standard deviation.

The groups were homogeneous regarding the type of insert used (CS: 24.8% vs. 21.6%, *p* = 0.70), gender (female: 57.5% vs. 52.9%, *p* = 0.61), age (69.7 vs. 68.7 years, *p* = 0.50), BMI (29.4 vs. 27.9 kg/m², *p* = 0.053), duration of final follow‐up (2.83 vs. 2.87 years, *p* = 0.39), preoperative KSS knee score (65.3 vs. 62.1; *p* = 0.06) and preoperative KSS function score (64.6 vs. 67.1; *p* = 0.41).

At the analysis of scores at the final follow‐up, a statistically significant difference was confirmed in terms of KSS function (*p* < 0.001) and KSS knee score (*p* = 0.04), as well as in ΔKSS knee (p < 0.001), in favour of the group with final rHKA90F ≥5° of varus. The difference between Groups A and B.2 exceeded the MCID in KSS function and ΔKSS knee. Detailed results are presented in Table 5.

**Table 5:** Comparison of scores at the final follow‐up between groups with final limb varus/valgus axis at 90° of flexion (rHKA‐90F) within 0–4° of varus (Group A) and those with a final rHKA‐90F ≥ 5° of varus (Group B.2).

	Group A (*N* = 113)	Group B.2 (*N* = 51)	*p*	MCID	Delta (A and B.2)
KSS knee	91.2 (SD 9.7)	94.8 (SD 5.6)	0.04	4.3	3.6
KSS function	89.7 (SD 11.3)	95.8 (SD 6.8)	<0.001	5.1	6.1
ΔKSS knee	24.8 (SD 16.5)	34.7 (SD 13.3)	<0.001	7.8	9.9
ΔKSS function	24.9 (SD 20.4)	29.1 (SD 18.9)	0.134	10.8	4.2
FJS‐12	74.2 (SD 21.7)	78.6 (SD 23.7)	0.117	11.2	4.4
AKPS	88.3 (SD 12.9)	89.7 (SD 15.8)	0.103	6.9	1.4

Abbreviations: AKPS, Kujala Anterior Knee Pain Scale; FJS‐12, Forgotten Joint Score; KSS, Knee Society Score; MCID, minimal clinically important difference; rHKA‐90F, robotic hip–knee–ankle angle at 90° of flexion; SD, standard deviation.

### Role of ΔrHKA‐90F in outcomes

The group with a ΔrHKA‐90F of ±2.5° (Group C) and the group with a ΔrHKA‐90F > 2.5° (Group D) were homogeneous regarding the type of insert used (CS: 29.1% vs. 19.4%, *p* = 0.15), gender (female: 57.0% vs. 53.7%, *p* = 0.75), age (69.4 vs. 69.4 years, *p* = 0.99), BMI (29.2 vs. 28.4 kg/m², *p* = 0.27), duration of final follow‐up (2.82 vs. 2.88 years, *p* = 0.51), preoperative KSS knee score (64.8 vs. 63.6; *p* = 0.54) and preoperative KSS function score (64.5 vs. 65.4; *p* = 0.74).

**Figure 4:**
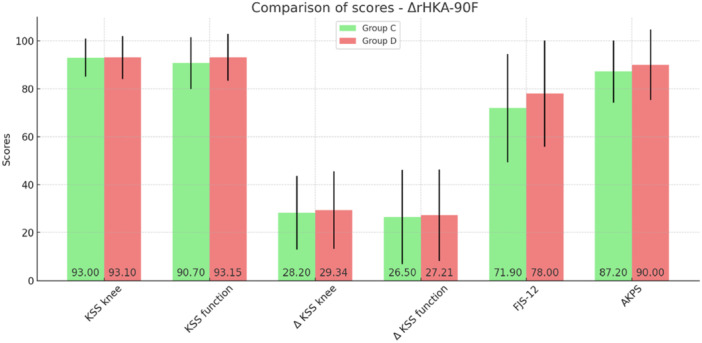
Chart of scores at the final follow‐up between groups with the difference between final and initial limb varus/valgus axis at 90° of flexion (ΔrHKA‐90F) of ±2.5° (Group C) and patients with a neutralized value (varus reduction or with a valgus value) greater than 2.5° (Group D). AKPS, Kujala Anterior Knee Pain Scale; FJS‐12, Forgotten Joint Score; KSS, Knee Society Score; rHKA‐90F, robotic hip–knee–ankle angle at 90° of flexion.

In the analysis of scores at the final follow‐up, a statistically significant difference was confirmed in terms of FJS‐12 (*p* = 0.041) and AKPS (*p* = 0.012), in favour of the group with ΔrHKA‐90F >2.5° of neutralization (varus reduction or with a valgus value). No differences between Groups C and D exceeded the MCID. Detailed results are presented in Table 6. The chart of the performed analysis is shown in Figure 4.

**Table 6:** Comparison of scores at the final follow‐up between groups with difference between final and initial limb varus/valgus axis at 90° of flexion (ΔrHKA‐90F) of ±2.5° (Group C) and patients with a neutralized value (varus reduction or with a valgus value) greater than 2.5° (Group D).

	Group C (*N* = 79)	Group D (*N* = 93)	*p*	MCID	Delta (C and D)
KSS knee	93.0 (SD 7.9)	93.1 (SD 9.0)	0.711	4.3	0.1
KSS function	90.7 (SD 10.8)	93.2 (SD 9.8)	0.095	5.1	2.5
ΔKSS knee	28.2 (SD 15.3)	29.3 (SD 16.2)	0.567	7.9	0.9
ΔKSS function	26.5 (SD 19.7)	27.2 (SD 19.1)	0.898	9.7	0.7
FJS‐12	71.9 (SD 22.5)	78.0 (SD 22.2)	0.041	11.2	6.1
AKPS	87.2 (SD 13)	90.0 (SD 14.7)	0.012	7.0	2.8

Abbreviations: AKPS, Kujala Anterior Knee Pain Scale; FJS‐12, Forgotten Joint Score; KSS, Knee Society Score; MCID, minimal clinically important difference; rHKA‐90F, robotic hip–knee–ankle angle at 90° of flexion; SD, standard deviation.

## DISCUSSION

The main findings of this study reveal significant predictors for the final rHKA‐90F and its clinical implications. The multiple linear regression analysis identified four key predictors of final rHKA‐90F: femoral rotation, tibial varus/valgus alignment, initial rHKA‐90F and final rHKA‐E. Another significant finding of this study is that the group with a final rHKA‐90F ≥ 5° of varus showed a statistically significant difference, with significantly higher values in KSS knee, KSS function and ΔKSS knee. KSS function and ΔKSS knee values also showed clinically significant differences. Additionally, in the analysis of ΔrHKA‐90F, a statistically significant difference was found in terms of FJS‐12 and AKPS, with better patient‐reported outcome measures (PROMs) observed in patients with a neutralized value (varus reduction or with a valgus value) greater than 2.5°. The role of femoral rotation and proximal tibial cuts on the gaps at 90°, and their consequent impact on the flexion axis, is validated by the fundamental principles of knee arthroplasty [2, 18, 30]. As is well known, the distal femoral cut and proximal tibial cut play a role in gap balancing in extension and affect the HKA. Similarly, femoral rotation and the proximal tibial cut influence gap balancing in flexion. This study further confirms their role in the coronal alignment in flexion.

Recent studies suggest that limb alignment in extension may not serve as a reliable indicator of alignment in flexion [24]. Relying exclusively on the traditional evaluation of coronal alignment in an extended lower limb for planning knee arthroplasty may therefore be insufficient.

Dynamic HKA alignment is a critical factor, as the knee is a moving joint, yet it often receives limited attention due to the lack of precise evaluation methods [19]. Computer navigation systems now enable the assessment of dynamic HKA alignment during surgery.

This study identifies initial rHKA‐90F, femoral rotation, tibial varus/valgus and final rHKA‐E as significant predictors of final rHKA‐90F, confirming that alignment is influenced by a combination of bony anatomy and soft tissue dynamics. As reported in previous studies, the kinematic behaviour of arthritic knees varies widely. It has been observed that the knee's functional flexion axis undergoes significant shifts in the frontal plane during motion from full extension to passive flexion at 120° [5]. Recognizing knee kinematic patterns before performing bone cuts or soft tissue releases may therefore assist in effectively managing flexion and extension gaps during TKA.

HKA alignment has been shown to change as the knee flexes from 0° to 90°, and proper alignment in full extension does not always ensure good alignment throughout flexion [32]. This variation is primarily influenced by the rotational alignment of the femoral component, with external rotation osteotomy of the distal femur playing a crucial role. The variation in HKA alignment during flexion tended to follow varus, valgus, or neutral patterns based on the selected angle of external rotation.

Dynamic imbalances may cause instability during movement, potentially leading to pain during activity and increased polyethylene wear, even in patients with a good mechanical axis in long‐leg standing alignment [10].

The results of this study provide further insights into the role of intraoperative rHKA‐90F as a predictor of clinical and functional outcomes in TKA. These results cannot be directly compared with the existing literature, as, to the best of our knowledge, no previous study has associated clinical outcomes with the axis at 90° of flexion. However, prior studies suggest that residual mild varus alignment (3–6°) is associated with superior functional outcomes compared to neutral alignment [16, 20, 25, 27, 31]. This could be attributed to the preservation of natural soft tissue tension, resulting in improved knee kinematics and patient satisfaction.

The positive results observed in Group B.2 may indicate a more natural kinematic pattern of the knee and enhanced soft‐tissue balance, even at 90° of flexion, supporting previous findings that slight varus alignment contributes to a more ‘natural feeling’ in the knee [7, 13, 27]. In fact, mild varus alignment has also been shown to reduce the risk of excessive medial soft tissue release, a common concern in patients with ‘constitutional’ varus [3, 15, 16].

Seemingly in contrast to these findings, avoiding excessive correction of rHKA‐90F (ΔrHKA‐90F < 2.5°), appears to be associated with better PROMs. This interpretation might be consistent with previous evidence suggesting that mild varus or neutral alignment yields better outcomes than severe varus alignment [20, 21, 23, 25, 29]. With regard to the axis in extension, it is also prudent to avoid overcorrection into valgus, which is associated with poorer outcomes [9].

While mild residual varus alignment has been associated with favourable outcomes, it is critical to avoid severe varus alignment (>6°), which has been linked to poorer functional and clinical results [20, 21].

Notably, this study confirms the deleterious effects of extreme deviations of alignment, highlighting the importance of a balanced strategy between accuracy of alignment and an individual‐specific approach. The significant differences observed in KSS knee and function scores and ΔKSS knee values between groups highlight the potential utility of rHKA‐90F as an intraoperative tool for predicting postoperative outcomes.

In fact, the apparent contradiction between a final rHKA‐90F ≥ 5° of varus, associated with significantly better KSS function and knee scores, and a ΔrHKA‐90F with a correction of >2.5° towards neutral alignment, associated with significantly better FJS‐12 and AKPS scores, is remarkable. This apparent contradiction highlights the importance of a personalized approach based on the patient's anatomy and kinematics, emphasizing the need for personalized alignment strategies. Osteoarthritic varus knees seem to achieve better functional outcomes when the degenerative deformity is corrected in flexion, while still requiring the preservation of a slight varus alignment. Soft tissues have to be part of the equation, and FA or FKP principles allow to consider both static (bone) and dynamic (soft tissue envelope) parameters not only in extension but also at 90° of flexion when personalizing implant positioning.

In light of these results, considering the coronal plane exclusively in extension, as is done in current classifications, could prove to be limiting. While promising, the results of this study highlight the need for further investigation to fully understand the implications of intraoperative dynamic alignment and its impact on the success of TKA.

### Limitations

Several limitations warrant consideration in this study. First, concerning the predictors for rHKA‐90F and ΔrHKA‐90F, the analysis did not account for the thickness of the bone resections. Instead, it focused exclusively on tibial and femoral varus/valgus angles, femoral flexion, femoral rotation and tibial slope, potentially overlooking critical variables that could influence alignment outcomes. The follow‐up duration, while sufficient for assessing short‐term outcomes, may not capture the long‐term effects of alignment strategies on implant survival, wear patterns or the development of complications. Furthermore, the influence of rHKA‐90F on other parameters, such as wear rates or joint stability, remains unexplored and warrants further investigation. Additionally, the study focused solely on patients with preoperative varus mHKA to maintain homogeneity. This choice is intended to reduce biases in the first analysis of this new parameter. While this approach strengthens internal validity, it may limit the applicability of findings to patients with preoperative valgus deformity or other alignment profiles. Although the measurement of alignment in flexion does not require varus–valgus stress forces, subjectivity may play a role, for example, in managing tibial rotation during the passive ROM. Moreover, objective biomechanical assessments or imaging follow‐ups, such as post‐operative stress radiographs or dynamic gait analysis, were not included, which might have provided more detailed insights into functional changes related to alignment. All patients were treated using the same CT‐based robotic system, the same type of implant and the same positioning principles. This may limit the extension of these results to all robot‐assisted TKA procedures. Finally, a more detailed analysis of variations of knee phenotypes should be conducted to obtain more personalized insights [4, 12].

## CONCLUSIONS

This study provides valuable insights into the predictors and clinical relevance of intraoperative rHKA‐90F in robotic TKA. The findings of superior clinical outcomes associated with a final rHKA‐90F ≥ 5° of varus and ΔrHKA‐90F with a correction of >2.5° towards neutral alignment can be interpreted as highlighting the importance of correcting arthritic varus deformity while avoiding neutral alignment and maintaining moderate varus, even in the flexion axis. Furthermore, these findings emphasize the importance of a personalized approach tailored to each patient's unique anatomy and kinematics.

## AUTHOR CONTRIBUTIONS

Luca Andriollo and Sebastien Lustig had the idea for the article. Luca Andriollo was responsible for writing the manuscript and qualified as the corresponding author. Pietro Gregori was responsible for data acquisition and analysis and realisation of figures and tables. Christos Koutserimpas was responsible for statistical analysis. Cécile Batailler and Elvire Servien were responsible for conceptualisation and supervised data acquisition and analysis. Cécile Batailler, Elvire Servien and Sebastien Lustig were responsible for reviewing and critically revising the manuscript. All authors have given final approval for the version to be published.

## CONFLICT OF INTEREST STATEMENT

Cécile Batailler is a consultant for Smith & Nephew and Stryker. Elvire Servien is a consultant for Smith & Nephew. Sébastien Lustig is a consultant for Heraeus, Stryker, DePuy Synthes, and Smith & Nephew. Additionally, their institution receives research support from Lepine and Amplitude. The remaining authors declare no conflicts of interest.

## ETHICS STATEMENT

This study adhered to the ethical principles outlined in the 1964 Declaration of Helsinki and complied with HIPAA regulations. Data collection and analysis were conducted in accordance with the MR004 Reference Methodology of the French Commission Nationale de l'Informatique et des Libertés (Ref. 2229975V0). All patients provided legitimate informed consent.

## Data Availability

The data that support the findings of this study are available from the corresponding author [L.A.], upon reasonable request.
